# Genetic Variation for Protein Content and Yield-Related Traits in a Durum Population Derived From an Inter-Specific Cross Between Hexaploid and Tetraploid Wheat Cultivars

**DOI:** 10.3389/fpls.2019.01509

**Published:** 2019-11-22

**Authors:** Angelica Giancaspro, Stefania L. Giove, Silvana A. Zacheo, Antonio Blanco, Agata Gadaleta

**Affiliations:** Department of Agricultural and Environmental Sciences (DiSAAT), Research Unit of “Genetics and Plant Biotechnology”, University of Bari Aldo Moro, Bari, Italy

**Keywords:** grain protein content (GPC), protein quality, quantitative trait loci (QTL), wheat, genetic map

## Abstract

Wheat grain protein content (GPC) and yield components are complex quantitative traits influenced by a multi-factorial system consisting of both genetic and environmental factors. Although seed storage proteins represent less than 15% of mature kernels, they are crucial in determining end-use properties of wheat, as well as the nutritional value of derived products. Yield and GPC are negatively correlated, and this hampers breeding programs of commercially valuable wheat varieties. The goal of this work was the evaluation of genetic variability for quantity and composition of seed storage proteins, together with yield components [grain yield per spike (GYS) and thousand-kernel weight (TKW)] in a durum wheat population obtained by an inter-specific cross between a common wheat accession and the durum cv. Saragolla. Quantitative trait loci (QTL) analysis was conducted and closely associated markers identified on a genetic map composed of 4,366 SNP markers previously obtained in the same durum population genotyped with the 90K iSelect SNP assay. A total of 22 QTL were detected for traits related to durum wheat quality. Six genomic regions responsible for GPC control were mapped on chromosomes 2B, 3A, 4A, 4B, 5B, and 7B, with major QTL on chromosomes 2B, 4A, and 5B. Nine loci were detected for GYS: two on chromosome 5B and 7A and one on chromosomes 2A, 2B, 4A, 4B, 7B, with the strongest QTL on 2B. Eight QTL were identified for TKW, three of which located on chromosome 3A, two on 1B and one on 4B, 5A, and 5B. Only small overlapping was found among QTL for GYS, TKW, and GPC, and increasing alleles coming from both parents on different chromosomes. Good candidate genes were identified in the QTL confidence intervals for GYS and TKW.

## Introduction

Mediterranean countries rely heavily on cereal production as main commodity crop of economic importance. Amongst cereals, durum wheat is the leading commercial crop as its consumption is the highest amongst Mediterranean countries. Given the always growing consumers' attention to health, objectives of wheat breeding programs are recently focusing not only on increasing productivity, but also on endowing derived products with a higher nutritional value (Battenfield et al., 2016). Wheat quality is a very complex issue including several components (Marcotuli et al., 2015). Usually, kernel protein quantity and quality are considered key factors of "wheat quality." Wheat quality, that is its ability to be processed into derived foods, is largely determined both by protein quantity—measured as grain protein content (GPC), and by protein quality—given by protein composition. Wheat proteins show high complexity and different interaction with each other, thus making them difficult to characterize. Wheat grain storage proteins are a complex mix of different polypeptide chains traditionally classified according to their solubility (following the sequential Osborne extraction procedure) or composition and structure (Zilic et al., 2011). Grain storage proteins include gliadin, glutenin, albumin, and globulin. In particular, gluten proteins that is gliadins and glutenins, give wheat flour unique extensibility and processing properties and consequently a good quality to derived products. Gluten is made up by proteins able to form a cohesive and visco-elastic dough by mixing flour with water. Differences in wheat processing properties are due to gluten quantity and composition. According to their solubility in aqueous alcoholic solutions, gluten proteins can be divided into soluble gliadins and insoluble glutenins. Gliadins are monomeric proteins responsible for gluten viscosity. Glutenins are polipeptidic aggregates responsible for dough strength and elasticity. After reduction of inter-molecular disulfide bonds, glutenin monomers are classified into high molecular weight (HMW) and low molecular weight (LMW) subunits (Payne et al., 1982; Jackson et al., 1983). Glutenin chains have high molecular weights up to tens of millions (Wrigley, 1996). Differences in glutenin subunits strongly influence end-use quality (Kasarda, 1999), in fact when managing the protein quality, one of the main aim of breeding programs is to select wheat varieties or lines that possess glutenin alleles associated with superior processing characteristics.

Wheat storage proteins are encoded by gene families with highly variable alleles. Gliadins are encoded by complex loci located on the short arms of homoeologous group 1 chromosomes (*Gli-A1*, *Gli-B1*, *Gli-D1* for omega and gamma) and 6 (*Gli-A2*, *Gli-B2*, *Gli-D2* for alpha- and beta-) (Shewry et al., 2003). For what concerns gutenins, LMW-GS derive from loci (*Glu-*3) on the short arms of group 1 chromosome (linked to *Gli-1* loci), whereas HMW-GS are coded by complex loci on the group 1 long arms (*Glu-1*). Each *Glu-1* locus contains two tightly linked paralogous genes encoding two different types of HMW-GS, namely the "x-" and a "y-" type, based on their electrophoretic mobility, with a wide combination of different alleles at each locus responsible for high polymorphism.

Hexaploid wheat contains six HMW-GS genes (*Glu-1*) located on 1A, 1B, and 1D chromosomes, whereas tetraploid wheat accounts for four HMW-GS loci on 1A and 1B. In hexaploid wheat there are five classes of HMW-GS proteins: 1Ax (encoded by *Glu-A1*),1Bx and 1By (encoded by *Glu-B1*), 1Dx and 1Dy (encoded by *Glu-D1*). The y-type genes at the *Glu-A1* locus are missing in hexaploid wheat (Galdi and Feldman, 1983). HMW glutenin-subunits from 1D are lacking in durum wheat. In hexaploid wheat, the 1Bx, 1Dx, and 1Dy HMW-GS are constitutively present in all cultivars, whereas 1Ay genes are always silent.

Gliadin and glutenins contribute together to determine wheat dough quality. In particular, the presence of some HMW-GS is crucial for the determination of wheat quality and its attitude to make pasta or bread (Jang et al., 2017). Noteworthy, 80% of the tetraploid genotypes miss the HMW-GS encoded by *Glu-A1* locus, with the consequence to be unsuitable for bread making quality (Branlard et al., 1989), in fact durum wheat is usually used to making pasta.

By influencing both the nutritional value and the processing properties of flour/semolina, GPC deeply contributes to determine the economic value of the crop. GPC is a typical quantitative trait controlled by several genes located on almost all chromosomes and influenced by both environment and management practices. As mature wheat grains usually contain 8 to 20% of proteins (Zilic et al., 2011), one of the main objectives of breeders is to identify stable QTL and relative favorable alleles, which can be successfully transferred from high-GPC donor lines to low-GPC varieties.(Kumar et al., 2018). However, reaching of this goal is hampered by the quantitative nature of the trait, which is controlled by multiple genes and strongly influenced by environmental factors and agronomical practices (Blanco et al., 2012). In fact, heritability for GPC has been reported to range from 0.41 (Kramer, 1979) to 0.70 (Suprayogi et al., 2009) depending on genotypes, location of field trials, and computational analysis.

Protein quality and quantity have always been among the crucial topics of wheat breeding. However, the increase of grain protein content is also complicated by its negative association with productivity, the strong environmental influence and the very narrow genetic variation found within the cultivated gene pool (Barneix, 2007; Iqbal et al., 2016). Modern wheat varieties are relatively low in GPC compared to their wild relatives, which could be employed as donors of useful alleles (Avivi, 1978; Nevo et al., 2002; Cakmak et al., 2004; Blanco et al., 2006; Chatzav et al., 2010). For example, wild emmer (*Triticum turgidum* ssp. *dicoccoides*) has a wide genotypic variation in several agronomic traits, including grain constituents (Uauy et al., 2006) and abiotic/biotic stress tolerance (Peleg et al., 2005; Ben-David et al., 2010). For this reason, the high genetic variability in the gene pool of wild emmer can be exploited, by crossing with durum wheat, for the improvement of several agronomic and economical important traits. However, its very low yield complicates wheat breeding for yield components.

Improvement of GPC by means of classical breeding methods have always given poor results. Recently, the release of genome sequences and comparative studies with model species (Colasuonno et al., 2013), and the consequent availability of advanced genetic tools such as linkage maps and molecular markers has fasten the improvement of GPC especially thanks to the application of marker-assisted selection (MAS) programs. Genetic factors and candidate genes influencing protein concentration in cultivated and wild wheats are located on all chromosomes. A recent review by Kumar et al. (2018) reported 49 QTL mapping publications with a total of 325 main effect QTL and 42 epistatic QTL for GPC.

Given the complexity of GPC genetic control and the existence of a very narrow genetic variation within cultivated gene pool, the improvement of this trait has always been a challenge for genetic breeders. Biodiversity is becoming an increasingly important issue in agriculture. Significant research of new valuable germplasm, breeding efforts, and subsequent plant multiplication are needed to improve the performance of cereal sector through better-suited varieties. It is fundamental continuing to achieve successful genotypes and to guarantee creation of new genetic diversity to allow a continued genetic gain in a dynamic agricultural environment.

Together with grain protein content, wheat yield is another complex agronomic trait resulting from the interaction of several components which are deeply influenced by environment. Understanding the molecular mechanisms underlying yield-related genes is one of the main objective of breeders. Several QTL have been mapped for some components such as thousand kernel weight, grain weight per spike, tiller number etc. (Huang et al., 2004; Narasimhamoorthy et al., 2006; Zhang et al., 2012), and some functional markers have been developed for thousand-kernel weight (TKW) (Su et al., 2011). However, gene isolation by map-based cloning is hampered by the hugeness and complexity of wheat genome, thus only few genes have been isolated through comparative genetics based on gene synteny (Su et al., 2011; Yang et al., 2012).

In the present work, an inter-specific cross between hexaploid and tetraploid wheat was used to map QTL for GPC and yield-related traits [grain yield per spike (GYS) and TKW], and identify putative candidate genes for the most associated markers. Moreover, the use of an accession of bread wheat as donor of useful genes for durum wheat, allowed to survey new genetic variability for grain quantity and composition, and identify new high quality lines useful in durum breeding programs.

## Materials and Methods

### Genetic Materials and Field Experiments

A total of 135 recombinant inbred lines (RILs) were obtained by the interspecific cross between the elite durum wheat cultivar Saragolla and the bread wheat accession 02-5B-318 (derived from the Chinese cv. Sumai-3) by advancing single F_2_ plants to the F_7_ by single seed descent (SSD) (Giancaspro et al., 2016). The cross produced two RIL populations: a sub-set of RILs were classified as hexaploid as carrying all the D chromosomes, and a sub-set of 135 durum RILs consisted of lines with no D chromosomes. In this work, only the durum RIL was taken into consideration for the study of grain protein content and yield-related traits The tetraploid lines were harvested, and seeds used for replicated field trials and DNA extraction. The parental lines for this interspecific cross were selected because different for several traits including yield components and protein content. The parents and the 135 RILs were evaluated for grain protein content and yield in replicated field trials at the location of Valenzano Bari-Italy for three years (2015–2017). The RILs were evaluated using a randomized complete block design with four replications. Each plots consisted of 1-m rows, 30 cm apart, with 80 seeds sown in each plot and supplemented with nitrogen (10 g/m^2^). Grain protein content was determined on a 2 gsample of whole meal flour by near-infrared reflectance (NIR) spectroscopy using a SpectraAlyzer device (Basic model, Zeuton). The instrument was calibrated by using a set of 25 whole-meal flour samples belonging to *T. turgidum* ssp. *durum*, ssp. *dicoccum*, and ssp. *dicoccoides* with known protein concentration and moisture, previously calculated according to official standard methods. Final GPC was expressed as a percentage of proteins on a dry weight basis. On each wheat line, GPC measurement was performed twice then the final value was averaged between the two technical replicates. GYS was measured as the total grain yield per row on the number of spikes per row (about 70–80 spikes). TKW was assessed from a 15 g seed sample per each plot (line). TKW of each wheat line was determined as the average of two technical replicates.

HMW-GS and gliadins analyses were performed as described by the method of Payne et al. (1980) and by Bushuk and Zillman (1978), respectively. The cv Chinese Spring of bread wheat and cv Svevo of durum wheat, containing known HMW-GS, were used as standards. HMW-glutenins composition was scored according to Payne's catalogue (Payne and Lawrence, 1983) that named HMW glutenins gene loci as *Glu-A1*, *Glu-1B*, and *Glu-1D* and proteins subunits as 0, 1, 2*, 2 + 12, 5 + 10, 6 + 8, 7 + 9, and 17 + 18.

### Genetic Map and QTL Analysis

A durum wheat genetic linkage map obtained by Giancaspro et al. (2016) was used for QTL analysis. The map, covering a total length of 4,227.37 cM, consists of 4,366 SNPs surveyed from the 81,587 sequences of the 90K iSelect array by Illumina CSProR (SanDiego, CA, USA) described by Wang et al. (2014).

ANOVA was conducted for each trait with standard procedures using X-Stat software and genetic variance (σ^2^_G_) and broad-sense heritability (h^2^_B_) calculated using the variance component estimates. Pearson phenotypic correlation coefficients were calculated by using M-STAT-C software between GPC, GYS, and TKW. The Inclusive Composite Interval Mapping (ICIM) method (Li et al., 2007) was used for QTL mapping with QGene 4.0 software (Joehanes and Nelson, 2008). Scanning interval of 2 cM between markers, and putative QTL with a window size of 10 cM was used for QTL detection. The number of marker cofactors for background control was set by forward regression with a maximum of five controlling markers. Putative QTL were defined as two or more linked markers associated with a trait at LOD ≥3. Positive and negative signs of the estimates QTL effect indicate the contribution of cv Saragolla and the 02-5B-318 accession, respectively. The phenotypic variance explained by each single QTL was quantified by the square of the partial correlation coefficient (R^2^). Graphical representation of linkage groups and QTL was obtained with MapChart 2.2 Software (Voorrips, 2002).

### HMW-GS Analysis

Wheat grains were grinded with porcelain mortar and pestle and boiled for 5' in an extraction buffer - ratio 10:1 (µl/g)—consisting of 0.4 ml β-mercaptoethanol, 4 ml pure water, and 1.7 ml γ-piromin dye (15 mg γ piromin; 2.0 g 100% SDS; 6.25 ml Tris-Hcl, pH 6.8; 10.5 ml water). β-mercaptoethanol served for the reduction of intermolecular disulphide bonds between glutenin subunits, SDS for protein denaturation, and γ-piromin for the visualization of protein bands on electrophoretic gel. Samples were incubated 2 h at room temperature, then centrifuged to recover supernatant. Electrophoresis was performed in SDS-PAGE with 4% stacking gel (pH 6.8) and 10% separating gel (pH8.8) for 24 h at 500 Volts, in a1X running buffer (Tris-SDS-Glycin, pH 8.3). Gels were left 24 h in a dying solution of Comassie Blue R250 composed by 12% tricloroacetic acid and 1%Comassie Brilliant Blue (19:1, v/v), then rinsed overnight with distilled water and visualized on UV. Common wheat cv. Chinese Spring and durum wheat cv. Svevo, with known HMW-GS, were used as standards.

### Gliadins Analysis

Gliadin extraction was performed on 30 µg of single-seed flour by following the protocol described in Bushuk and Zillman (1978). 100 µl of 70% ethanol were added to flour and incubated at 24°C for 1 h with brief vortexing at 10 min intervals. Supernatant was collected by centrifuging tubes at 12,000 rpm for 10 min at room temperature. Then, 26 µl of gel buffer was added to 20 µl of supernatant and loaded onto the gel for PAGE separation. Details on gel preparation and run are described in Bushuk and Zillman (1978). The electrophoregrams were evaluated based on gliadin relative mobility.

### Candidate Gene Analysis

In order to assign genes to SNPs, sequences corresponding to all the SNP markers mapping in the confidence intervals of QTL for GPC, GYS and TKW were used in a BLAST search using TBLASTX algorithm (http://ncbi.nlm.nih.gov/BLAST), against the wheat draft genome sequence of the tetraploid *dicoccoides* accession Zavitan (Avni et al., 2017), the durum cv. Svevo (Maccaferri et al., 2019), and the hexaploid Chinese Spring (Appels et al., 2018) in order to assign a putative function to SNPs, and identify candidate genes (CGs) for the traits of interest. Alignments with at least 80% identity and E-value >10^-7^ were considered, and the corresponding putative genes evaluated for their involvement in metabolic pathways related to protein content and yield components.

Molecular Structure (Exons/Introns) and Function (Corresponding Coding Protein) Was Determined for the Most Associated CG to TWK QTL: Elongation Factor (EF) Gene. Comparison Was Carried on Among Diploid (*Triticum Urartu*), Tetraploid (*T. Durum*, Cv. Svevo, *T. Dicoccoides*), and Hexaploid (*T. Aestivum*, Cv. Chinese Spring) Wheats. Molecular Characterization Was Obtained by Browsing the Corresponding SNP Sequence (IWB30162, Excalibur_Rep_C106003_475) in the Svevo Genome Browser at: https://Interomics.Eu/Durum-Wheat-Genome and in Plant Ensemble Databases (https://Plants.Ensembl.Org).

## Results

### Field Traits Analysis

**Figure 1:**
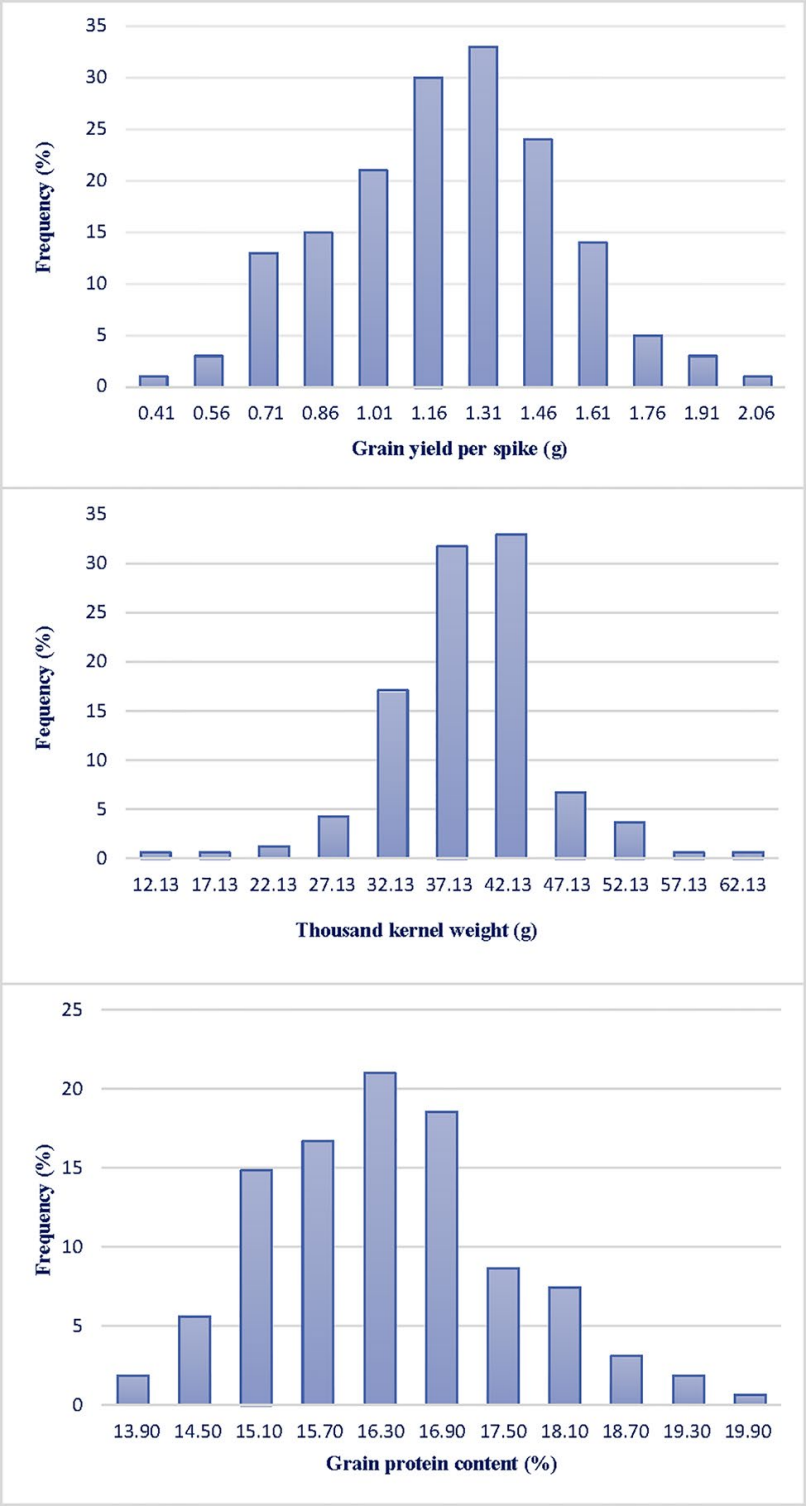
Frequency distribution of grain yield per spike (GYS), thousand kernel weight (TKW) and grain protein content (GPC) in a RIL population derived by the cross between the durum cv Saragolla and the bread wheat accession 02-5B-318. Traits were evaluated at Valenzano (Bari, southern Italy) for three years (2015, 2016, 2017); the mean value of the three years are presented.

GYS and TKW were evaluated at Valenzano (Bari) in southern Italy for three years (2015, 2016, 2017). **Table 1** reports the mean value and the range of GPC and yield components for parents Saragolla and 02-5B-318 and for RI lines, together with variance components and broad-sense heritability estimates, in each of the three environments. GPC values were different between the two parents both in Valenzano 2015, 2016, and 2017, with a mean value of 14.23 for cv Saragolla and 13.56 for 02-5B-318. As expected for a typical quantitative trait, GPC values showed a broad variability across the RI lines in each of the three environments, in fact the phenotypic means of RILs were distributed across a normal curve (**Figure 1**). The RIL population means (16.4, 16.7, and 15.0 for the three years, respectively), were significantly higher of both mid-parental values and with a minimum value of 12.8 and a maximum value of 22.10 recorded at Valenzano 2015. Differences in mean values and variances of parental lines and RI population observed in the different locations, were very likely due to the different environmental factors. Broad sense heritability (genotype mean basis) of GPC ranged from 0.47 to 0.48 in the three environments.

**Table 1:** Means, ranges, coefficients of variation (CV), genetic variance (σ^2^_G_), and heritability (h^2^_B_) of grain protein content and grain yield components in the Saragolla x 02-5B-318 RIL population and parental lines, evaluated in three environments.

Trait	Environments			
	Valenzano 2015	Valenzano 2016	Valenzano 2017	Mean
**Grain Protein Content, GPC (%)**				
02-5B-318	13.88	14.45	12.37	13.56
Saragolla	12.33	15.12	13.25	13.56
Mean RIL	16.37	16.66	15.06	16.03
Range	12.8-22.10	13.44-21.80	12.41-18.12	12.88-20.67
CV(%)	6.66	6.50	5.90	6.35
σ^2^_G_	1.04	1.00	1.10	1.04
h^2^_B_	0.48	0.47	0.47	0.47
**Grain Yield per Spike, GYS (g)**				
02-5B-318	1.30	1.47	0.99	1.25
Saragolla	2.26	2.78	1.68	2.24
Mean RIL	1.13	1.26	1.05	1.15
Range	0.22-2.40	0.38-2.45	0.47-1.88	0.36-2.24
CV(%)	20.88	19.90	20.00	20.26
σ^2^_G_	0.08	0.07	0.08	0.08
h^2^_B_	0.57	0.50	0.52	0.53
**1000 Kernel Weight, TKW (g)**				
02-5B-318	32.68	43.56	30.58	35.61
Saragolla	41.76	51.52	38.16	43.81
Mean RIL	35.71	36.32	36.55	36.19
Range	22.51-67.84	15.50-60.41	9.93-53.00	15.98-60.41
CV(%)	11.01	12.00	11.92	11.64
σ^2^_G_	35.18	34.20	36.00	35.13
h^2^_B_	0.69	0.68	0.69	0.62

Saragolla and 02-5B-318 showed a significant difference also for the yield components GYS and TKW; consequently, a large variation was observed for these two traits in the RIL population in all the three environments. The durum cv. Saragolla showed higher values in each environment for both traits: in particular, the mean values for GYS and TKW were 2.24 and 43.81 g, respect to 1.14 and 35.60 g of the same traits in 02-5B-318 parent. Interestingly, for both GYS and TKW, the trait variation was much larger in the RILs population than between the parental lines, with lowest values of 0.22 and 9.93 g and highest values of 2.45 and 67.84, for GYS and TKW, respectively, which suggested the presence of favorable alleles increasing the trait in both parents. For example, in all the three environments, the difference between the highest and the lowest value of GYS in the RI lines was more than twice the difference between the two parents. Heritability across environments was high for both GYS (mean value of 0.53) and TKW (mean value of 0.69).

As expected, in the present work we found a negative correlation between GPC and yield components (**Table 2**). In particular, GPC resulted negatively correlated with GYS (r values ranging from -0.09 to -0.16) in all environments, and with 1,000 kernel weight only at Valenzano 2017 (r = -1.00).

**Table 2:** Correlation coefficients between grain protein content and grain yield components in the Saragolla x 02-5B-318 RIL mapping population, evaluated in three environments. (Valenzano 2015, Valenzano 2016, Valenzano 2017).

Trait	Grain protein content		
	Valenzano 2015	Valenzano 2016	Valenzano 2017
Grain yield per spike	–0.11***	–0.09***	–0.16***
1000 kernel weight	–0.07	0.43	–1.00*

*,*** Significant differences at 0,05 P, 0,01 P e 0,001 P respectively.

### QTL Analysis

**Figure 2:**
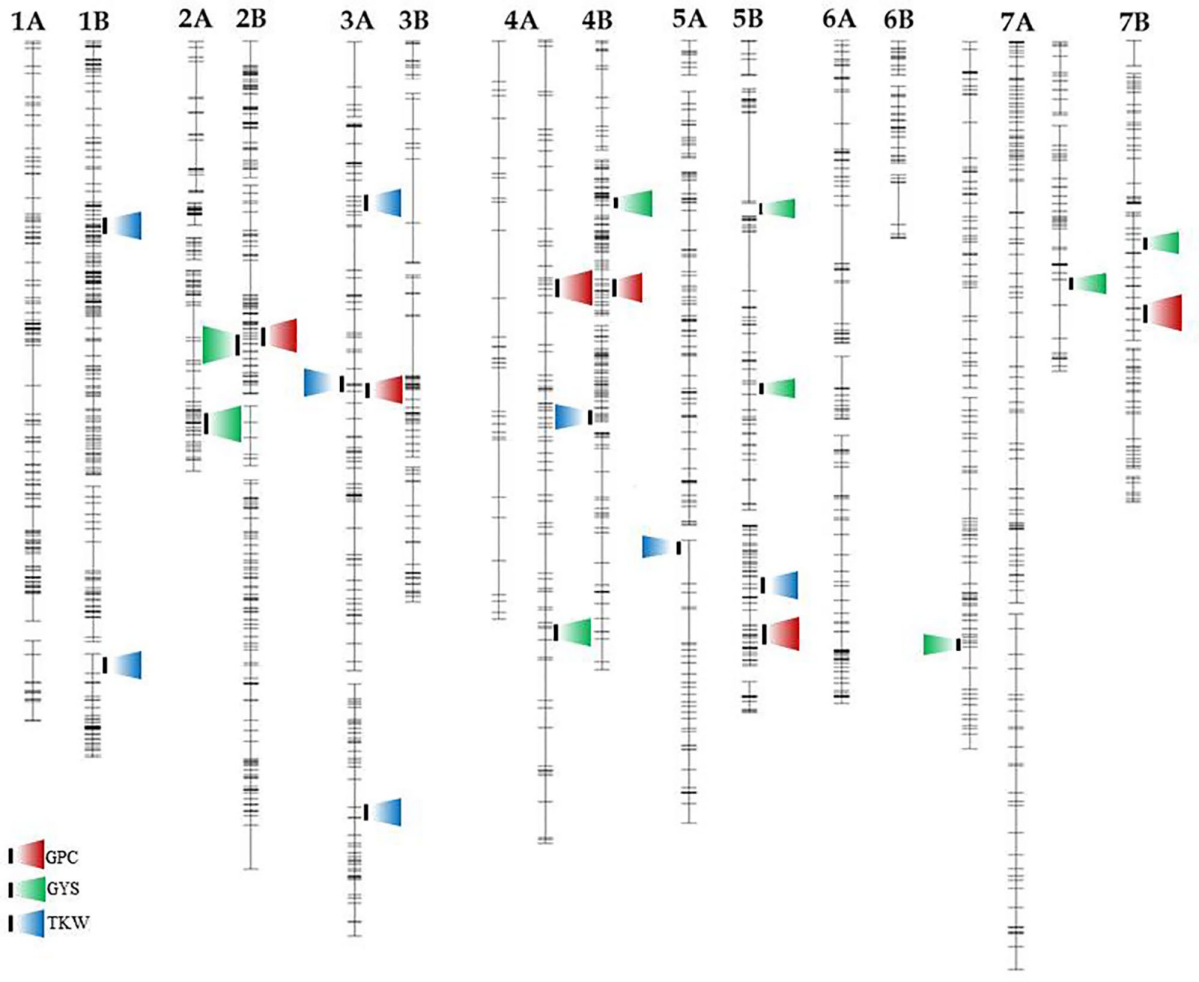
Genetic location of QTL for GYP, TKW, and GPC mapped on the durum wheat linkage map obtained in the RIL population derived by the cross between the durum cv. Saragolla and the bread wheat accession 02-5B-318. Loci for the different traits are reported in different colors (GPC, red; GYS, green; TKW, blue).

QTL analysis was performed according to the Inclusive Composite Interval Mapping method (ICIM, Li et al., 2007). Putative QTL (LOD ≥ 3.0) for GPC, GYS, and TKW in individual environments are listed in **Tables 3**–**5** respectively. QTL positions on the durum wheat linkage map are reported in **Figure 2**. Suggestive QTL, with a LOD value comprised between 2.0 and 3.0, were also reported.

**Table 3:** QTL for grain protein content (GPC) mapped in the durum wheat RIL population derived from the cross between the bread wheat accession 02-5B-318 and the durum wheat cv. Saragolla, evaluated in three different environments (Valenzano 2015, Valenzano 2016, Valenzano 2017). Analyses were performed by ICIM (Inclusive Composite Interval Mapping).

Chrom.	Linkage group	Marker interval	Most associated marker	Map position (cM)	Valenzano 2015			Valenzano 2016			Valenzano 2017			Environment mean		
					Add ^a^	LOD	R^2^	Add	LOD	R^2^	Add	LOD	R^2^	Add	LOD	R^2^
**2BS**	2B-2	IWB31001-IWA897	IWB72906	67.6	–0.7	5.6	20	–0.4	2.2°	08	–0.3	2.4°	09	–0.4	4.9	17
**3AS**	3A-2	IWB64668-IWB72529	IWB72484	108.5	–0.4	2.4°	09	–0.4	3.3	12	–	–	–	–0.3	2.2°	08
**4AL**	4A-2	IWB7798-IWB39495	IWB54916	76.3	–.6	4.1	15	0.6	6.4	22	–	–	–	–.4	5.6	19
**4BL**	4B-2	IWB36086-IWB34895	IWB55835	58.6	–0.6	3.8	14	–	–	–	–	–	–	–0.4	4.3	15
**5BL**	5B-4	IWB54873-IWB11747	IWB11571	31.6	0.7	6.2	22	0.5	3.7	13	0.4	5.5	19	0.5	7.0	23
**7BL**	7B-3	IWB10498-IWB69574	IWB69002	39.0	0. 6	3.9	15	0.4	3.2	12	–	–	–	0.4	4.4	15

° Suggestive QTLs at the sub-threshold 2.0 < LOD < 3.0.

- Not significant QTL.

a Additive Effect: positive additive effects are associated with an increased effect from Saragolla alleles and negative additive effects with an increased effect from 02-5B-318 alleles

R^2^: Percentage of phenotypic variance explained by the additive effects of the mapped QTL.

**Table 4:** QTL for grain yield per spike (GYS) mapped in the durum wheat RIL population derived from the cross between the bread wheat accession 02-5B-318 and the durum wheat cv. Saragolla, evaluated in three different environments (Valenzano 2015, Valenzano 2016, Valenzano 2017). Analyses were performed by ICIM (Inclusive Composite Interval Mapping).

Chrom.	Linkage group	Marker interval	Most associated marker	Map position (cM)	Valenzano 2015			Valenzano 2016			Valenzano 2017			Environment mean		
					Add ^a^	LOD	R^2^	Add	LOD	R^2^	Add	LOD	R^2^	Add	LOD	R^2^
**2AL**	2A-3	IWA32-IWB71282	IWB7315	6.9	0.1	6.2	21	0.2	7.7	27	0.1	3.6	13	0.1	10.4	32
**2BS**	2B-2	IWA1665-IWA897	IWB320054	67.0	0.3	23.6	29	0.3	3.3	12	0.3	23.0	29	0.3	23.6	08
**4AL**	4A-2	IWB87-IWB12722	IWB59450	170.7	–0.1	5.1	18	–	–	–	–	–	–	–0.1	3.7	13
**4BL**	4B-2	IWB71836-IWB70999	IWB64615	13.3	0.1	6.7	23	–	–	–	–	–	–	–0.1	5.2	18
**5BL**	5B-2	IWB72334-IWB20927	IWB72334	45.3	–0.1	4.8	17	–	–	–	–	–	–	–0.1	4.2	15
**5BL**	5B-3	IWA8097-IWB149734	IWB34530	66.7	–0.1	5.1	18	–	–	–	–	–	–	–	–	–
**7AL**	7A-6	IWB7367-IWA8312	IWA6576	72.8	0.1	3.9	14	–	–	–	–	–	–	0. 8	3.2	11
**7BL**	7B-3	IWB1711-IWB9018	IWA8570	16.7	–0.1	6.1	21	–	–	–	–0.1	5.4	19	–0.1	8.0	26

- Not significant QTL.

a Additive Effect: positive additive effects are associated with an increased effect from Saragolla alleles and negative additive effects with an increased effect from 02-5B-318 alleles

R^2^: Percentage of phenotypic variance explained by the additive effects of the mapped QTL.

**Table 5:** QTL for thousand kernel weight (TKW) mapped in the durum wheat RIL population derived from the cross between the bread wheat accession 02-5B-318 and the durum wheat cv. Saragolla, evaluated in three different environments (Valenzano 2015, Valenzano 2016, Valenzano 2017). Analyses were performed by ICIM (Inclusive Composite Interval Mapping).

Chrom.	Linkage group	Marker interval	Most associated marker	Map position (cM)	Valenzano 2015			Valenzano 2016			Valenzano 2017			Environment mean		
					Add^a^	LOD	R^2^	Add	LOD	R^2^	Add	LOD	R^2^	Add	LOD	R^2^
**1BS**	1B-1	IWB62561-IWB8572	IWB10407	72.0				–2.4	4.7	17						
**1BL**	1B-3	IWB69144-IWB14436	IWB69144	0.00	–2.1	3.6	13	–3.6	9.6	32	–2.0	4.2	15	–2.3	5.9	20
**3AS**	3A-2	IWA950-IWB73247	IWB73711	37.2	3. 6	8.2	27	2.8	6.0	21	2.9	7.4	25	3.3	10.3	32
**3AS**	3A-2	IWB71453-IWB27100	IWB8477	91.6	-2.8	4.9	18	–3.0	6.1	22	–3.6	10.1	32	–3.1	8.5	27
**3AL**	3A-3	IWB58806-IWB70483	IWB37509	49.7	–	–	–	–2.2	3.8	14	–	–	–	–1.6	2.9°	11
**4BL**	4B-3	IWB7164-IWB24289	IWA892	33.0	–	–	–	–3.0	6.6	23	–	–	–	–	–	–
**5AL**	5A-4	IWA1258-IWB72888	IWA1258	0.00	–	–	–	–2.1	3.3	13	–	–	–	–	–	–
**5BL**	5B-4	IWB42947-IWB764	IWB7719	15.9	-4. 8	5.1	28	-5.7	5.8	28	-4.5	46.2	83	-4.8	5.5	28

° Suggestive QTLs at the sub-threshold 2.0 < LOD < 3.0.

- Not significant QTL.

a Additive Effect: positive additive effects are associated with an increased effect from Saragolla alleles and negative additive effects with an increased effect from 02-5B-318 alleles

R^2^: Percentage of phenotypic variance explained by the additive effects of the mapped QTL.

In the present work we identified six major QTL for GPC, each on chromosome 2B, 3A, 4A (two loci), 4B, 5B, and 7B, responsible for the highest percentage of the overall phenotypic variation of the trait in the three environments. Interestingly, we found trait-increasing alleles coming from both the high-GPC parent Saragolla and from the low-GPC parent 02-5B-318. Specifically, alleles from Saragolla were those mapped on chromosomes 4A, 5B, and 7B, whereas alleles from 02-5B-318 were those on 2B, 3A, and 4B. The additive effect values ranged from 0.27 to 0.71 of protein content unit. The percentage of phenotypic variation (R^2^) contributed by each single QTL in each environment was comprised between 8 and 23%. QTL on 2B and 5B were found significant in all the environments, whereas three were significant in two environments, and one was significant in one single location (**Table 3**).

Eight chromosomal regions were detected for GYS (**Table 4**), two on chromosome 5B and 7A and one on chromosomes 2A, 2B, 4A, 4B, 7B. Five QTL came from cv Saragolla and four from the 02-5B-318 accession line, three of which were found in all the environments. The strongest QTL was the one detected on 2B chromosome with a LOD score of 23; this stable QTL was responsible of up to 29% of GYS variation, and the positive allele came from Saragolla, the durum parental line with the highest GYS. The GYS QTL on 7B was significant in two environments, and the positive allele came from 02-5B-318. The remaining five QTL mapped for GYS were significant only in one single environment, with a quote of phenotypic variation comprised between 14 and 23%.

In the present work we mapped eight QTL for TKW, three of which were located on chromosome 3A, two on 1B and one on 4B, 5A, and 5B (**Table 5**). Four QTL were stable in the three environments and significant in the mean of environments, while the others QTL were significant only in one single environment. Each QTL had an additive effect ranging from of 1.67 g (QTL on 3A-3) to 5.67 g (QTL on 5B), and explained 11 to 32% of trait variation. Out of the eight QTL detected, six came from the bread parental line accession 02-5B-318, whereas only two derived from the durum wheat cv Saragolla.

Interestingly, only few overlapping occurred between QTL for grain yield components and QTL for GPC on 2B and 3A chromosomes (**Figure 2**). In most cases, the QTL was responsible for the genetic control of only one of the traits.

### Putative Function and Candidate Gene Detection for GPC and Yield Components

One of the major steps of a gene-based analysis is the assignment of SNPs to genes. In order to identify a putative function for SNP markers and search for CGs for the traits of interest, SNP sequences were BLAST searched against GeneBank non redundant (nr) database. A total of 378 SNP sequences included in QTL regions were analysed, specifically 83 for GPC, 129 for TKW and 166 for GYP (for each QTL, SNPs from co-migrating loci mapped in a confidence interval of 15 cM were considered). **Tables 6**–**8** list SNP loci for which sequence homology was retrieved for GPC, GYS and TKW, respectively. Overall, 22% of the total query sequences matched with known function genes (E value > 10^-7^), but only 10% corresponded to protein products involved in metabolic pathways related to the traits of interest. Sequences similarity searches revealed storage proteins loci, and genes involved in different cellular processes such as: DNA transcription, chromatin structure determination, response to biotic and abiotic stress, carbon metabolisms, modification of cell wall structure, and intracellular signalling.

**Table 6:** Candidate genes identified in the QTL for grain protein content (GPC) mapped in the interspecific RIL population derived by the cross between the hexaploid wheat accession 02-5B-318 and the durum wheat cv. Saragolla (putative function is reported for the SNP markers in the QTL confidence interval showing an identity percentage > 80%).

QTL confidence interval	SNP name	SNP id	Chromosome arm	Linkage Group	Map position (cM)	Putative candidate gene	Species	E-value	Identity percentage (%)
IWB31001-IWA897	GENE-0644_370	IWB32005	2BS	2B-2	67.1	E3_Ubiquitin_protein ligase (SP1)	*B. distachyon*	3e^-30^	89.8
	Excalibur_c1305_662	IWB22202	2BS	2B-2	67.1	E3_Ubiquitin_protein ligase (SP1)	*B. distachyon*	3e^-30^	89.8
	GENE-0644_421	IWB32007	2BS	2B-2	67.1	E3_Ubiquitin_protein ligase (SP1)	*B. distachyon*	3e^-30^	89.8
	Ra_c106376_879	IWB50813	2BS	2B-2	67.1	RNA ligase isoform 2	*T. aestivum*	8e^-44^	99.0
IWB64668-IWB72529	RFL_Contig4403_1034	IWB64668	3AS	3A-2	105.4	Serine carboxypeptidase-like 33 (GS5)	*T. aestivum*	8e^-45^	99.0
	wsnp_Ex_c10272_16842803	IWA1308	3AS	3A-2	105.4	Serine carboxypeptidase-like 33 (GS5)	*T. aestivum*	8e^-45^	99.0
	Excalibur_c8930_548	IWB29267	3AL	3A-2	119.6	Tryptophan aminotransferase related 3 (TAR3.1-3B)	*T. aestivum*	6e^-15^	100.0
IWB36086-IWB34895	IAAV8654	IWB35513	4BL	4B-2	55.7	GW2-B gene, promoter region	*T. turgidum* ssp. *durum*	1e^-28^	87.6
IWB10498-IWB69574	BS00070791_51	IWB10498	7BL	7B-3	34.7	Omega/gamma/delta gliadin (LMW-D1/D2/D3/D6/D7)	*T. aestivum*	9e^-10^	78.2
	Tdurum_contig23504_196	IWB69002	7BL	7B-3	39.0	Omega/gamma/delta gliadin (LMW-D1/D2/D3/D6/D7)	*T. aestivum*	9e^-10^	78.2
	Excalibur_c57808_355	IWB27761	7BL	7B-3	39.1	Omega/gamma/delta gliadin (LMW-D1/D2/D3/D6/D7)	*T. aestivum*	9e^-10^	78.2
	Tdurum_contig28630_245	IWB69574	7BL	7B-3	43.6	PDI-like protein (pdil8-1)	*T. aestivum*	3e^-06^	88.1

**Table 7:** Candidate genes identified in the QTL for grain yield per spike (GYS) mapped in the interspecific RIL population derived from the cross between the hexaploid wheat accession 02-5B-318 and the durum wheat cv. Saragolla (putative function is reported for the SNP markers in the QTL confidence interval showing an identity percentage > 80%).

QTL confidence interval	SNP name	SNP id	Chromosome arm	Linkage Group	Map position (cM)	Putative candidate gene	Species	E-value	Identity percentage
IWA1665- IWA897	BS00083078_51	IWB11285	2BS	2B-2	62.2	Gamma gliadin-A1/A3/A4 and LMW-A2 genes	*T. aestivum*	3e^-12^	85.7
	JD_c39990_130	IWB37419	2BS	2B-2	64.2	Gamma gliadin-A1/A3/A4 and LMW-A2 genes	*T. aestivum*	3e^-12^	85.7
	Tdurum_contig62595_466	IWB72906	2BS	2B-2	67.7	Secologanin synthase (Cyt P450)	*Ae. tauschii*	5e^-37^	94.1
IWB71836-IWB70999	RFL_Contig4212_597	IWB64614	4BS	4B-2	13.6	Pyrroline 5-carboxylate synthetase (P5CS1)	*T. turgidum* ssp. *durum*	8e^-15^	80.2
	Tdurum_contig98478_400	IWB74042	4BS	4B-2	13.8	Alcohol dehydrogenase (ADH1A)	*T. turgidum* ssp. *dicoccoides*	1e^-06^	100.0
	Tdurum_contig98478_494	IWB74043	4BS	4B-2	13.8	Alcohol dehydrogenase (ADH1A)	*T. turgidum* ssp. *dicoccoides*	1e^-06^	100.0
IWB72334-IWB20927	wsnp_Ku_c14202_22436656	IWA6516	5BL	5B-2	46.2	Gamma,delta,omega gliadin	*T. aestivum*	1e^-13^	80.9
	Tdurum_contig11060_433	IWB66909	5BL	5B-2	46.2	Gamma,delta,omega gliadin	*T. aestivum*	1e^-13^	80.9
IWA8097-IWB14973	IAAV2296	IWB34530	5BL	5B-3	66.7	Transparent testa glabra1 *(TTG-1*)	*Ae. tauschii*	3e^-30^	95.0

**Table 8:** Candidate genes identified in the QTL for thousand kernel weight (TKW) mapped in the interspecific RIL population derived by the cross between the hexaploid wheat accession 02-5B-318 and the durum wheat cv. Saragolla (putative function is reported for the SNP markers in the QTL confidence interval showing an identity percentage > 80%).

QTL confidence interval	SNP name	SNP id	Chromosome arm	Linkage Group	Map position (cM)	Putative candidate gene	Species	E-value	Identity percentage
IWB62561- IWB8572	Excalibur_c20610_149	IWB23524	1BS	1B-1	66.9	Annexin 6-2	*T. turgidum* ssp. *Durum*	2e^-21^	98.4
	wsnp_BF291549B_Ta_1_1	IWA435	1BS	1B-1	66.9	GSK-like kinase 1B (GSK1B)	*T. aestivum*	2e^-53^	83.2
	Excalibur_c20610_251	IWB23525	1BS	1B-1	66.9	Annexin 6-2	*T. turgidum* ssp. *Durum*	2e^-21^	98.4
	IAAV2366	IWB34541	1BS	1B-1	75.1	Gamma-glutamylcysteine synthetase (GSH1)	*T. aestivum*	9e^-56^	99.0
IWB69144- IWB14436	CAP7_rep_c6352_375	IWB14436	1BL	1B-3	11.8	Chlorophyll a-b binding protein of LHCII	*Ae. Tauschii*	7e^-41^	97.1
IWA950- IWB73247	CAP8_c2839_118	IWB14646	3AS	3A-2	20.3	GID2-A1 protein (gid2-A1)	*T. aestivum*	5e^-35^	98.9
	RAC875_c64107_404	IWB59845	3AS	3A-2	42.0	Gamma gliadin-A	*T. aestivum*	7e^-08^	75.5
IWB58806- IWB70483	Tdurum_contig34075_98	IWB70483	3AL	3A-3	54.2	Gamma/delta/omega gliadin-B	*T. aestivum*	7e-08	75.5
IWB7164- IWB24289	CAP8_rep_c3658_272	IWB15007	4BL	4B-3	35.6	Catalase (CAT)	*T. turgidum* ssp. *durum*	1e^-42^	99.0
IWA1258- IWB72888	Tdurum_contig9074_2085	IWB73761	5AL	5A-4	17.6	RNA helicase (DEAD1-B)	*T. aestivum*	1e^-18^	87.6
IWB42947- IWB764	Excalibur_rep_c106003_475	IWB30162	5BL	5B-4	11.0	Protein elongation factor	*T. aestivum*	7e^-43^	99.0
	Excalibur_c9370_944	IWB29437	5BL	5B-4	11.9	Squamosa promoter-binding-like protein 6 (SPL6)	*T. aestivum*	9e^-04^	73.3
	BobWhite_c15241_604	IWB764	5BL	5B-4	20.9	Ammonium transporter (AMT2.1)	*T. aestivum*	6e^-40^	97.0

As reported in **Table 6**, putative functions retrieved for SNPs included in the GPC-QTL were seed storage proteins (ω/γ/δ gliadins), or enzymes involved in RNA processing (RNA ligase), amino acid modification (serine carboxypeptidase, tryptophan aminotransferase), determination of protein tertiary structure (PDI-like protein) and modification of proteins destined to degradation (Ubiquitin_protein ligase, GW2-B gene promoter).

**Table 7** shows the putative function detected for the SNP markers mapped in the GYS-QTL. Some sequences matched with genes for gliadin storage protein; other SNPs were found to fall into genes related to plant response to stress (Secologanin synthase - Cyt P450- and Pyrroline 5-carboxylate synthase) or involved in sugar metabolism (Alcohol dehydrogenase, ADH1A). In this work, a particular attention was paid to the candidate gene matching with the marker IAAV2296 (IWB34530). Bioinformatics research showed that SNP sequence has a high similarity percentage (95%) with the *Aegilops tauschii* gene for *Trasparent-testa-glabra1* (*TTG-1*). Molecular structure of *TTG-1* was compared in diploid (*T. urartu*, *Aegilops tauschii*), tetraploid (*T. durum*, cv. Svevo, *T. dicoccoides*), and hexaploid (*T. aestivum*, cv. Chinese Spring) wheats, where genes were located on A, B, and D genome. Details on gene structure of *TTG* in wheat, together with *Arabidopsis*, tobacco and rice are reported in **Supplementary Table S2**.

For what concerns TKW, the identified candidate genes in the QTL region were storage ω/γ/δ gliadins, RNA helicase, proteins involved in photosynthesis (**Table 8**). Attention was focused on candidate genes identified in the confidence interval for the QTL on 5B chromosome, where major QTL for TKW have been cited in literature (Wang et al., 2012; Assanga et al., 2017; Su et al., 2018). In this region, we found very good candidates as some of the SNP markers fell into genes in some way involved in the determination of kernel weight, such as a protein elongation factor (Excalibur_rep_c106003_475), a squamosa promoter-binding-like protein (Excalibur_c9370_944), and an ammonium transporter (BobWhite_c15241_604). Among these, we decided to better characterize the EF gene, whose homologous on 7A chromosome has recently (Zheng et al., 2014) been assessed to be significantly associated with grain number per spike and to potentially increase wheat grain yield and yield-related traits. The molecular structure was compared in diploid (*Triticum urartu*), tetraploid (*T. durum*, cv. Svevo, *T. dicoccoides*), and hexaploid (*T. aestivum*, cv. Chinese Spring) wheats (**Supplementary Table 1**). The gene could be identified only on 5B and 5D homoeologous chromosomes, whereas no genes were found on A genome, in both tetraploid and hexaploid genotypes. *EF* gene is characterized from having several alternative splicing forms, with a variable number of exons/introns.

### Grain Protein Composition

In the present work both parents and the complete RIL population were characterized for HMW-GS and for the gliadin *Gli-B1* locus (**Table 9**). For HMW-GS the bread parent has for *Glu-A1* gene the 1Ax2* allele encoding for 2* HMW-GS, for *Glu-B1* the *Bx7* and *By9* alleles encoding 7 + 9 HMW-GS and γ42 gliadin subunit for *Gli-B1* gene, while the durum wheat cv Saragolla presented the following allele combination: 6 + 8 for HMW-GS and the γ-45 for *Gli-B1* gene. A segregation for all genes, reported in **Table 9**, was observed in the RIL population, and in particular on 135 analyzed RI lines, 72 showed the HMW-GS profile of Saragolla parent (6 + 8) and 63 RI lines the HMW-GS of the bread wheat parent (7 + 9). Moreover, 55 RI lines showed the γ-45 gliadin allele coming from cv Saragolla and 80 the γ-42 allele of the bread wheat parent.

**Table 9:** HMW-GS and γ-42/γ-45 gliadin segregation in the durum wheat RIL population derived from the cross between the bread wheat accession 02-5B-318 and the durum cv. Saragolla.

	Glu-A1		Gli-B1		Gli-B1	
	2*	null	7+9	6+8	γ-42	γ-45
**02-5B-318**	+	–	+	–	+	–
**Saragolla**	–	+	–	+	–	+
**N. of RILs**	3	132	72	63	55	80

## Discussion

In the present work, an inter-specific RIL population of 135 tetraploid lines, originally developed by Giancaspro et al. (2016) for investigating the genetic basis of Fusarium head blight (FHB) resistance in durum wheat, was further characterized for GPC, protein composition, and yield components (TKW and GYS). In all the environments, the traits showed a wide difference between the two parents and a big variability in the RIL population. In particular, the range of phenotypic values in the RIL population was larger than between the two parents, clearly indicating the establishment of new genetic variability in the RI lines due to the combination of favorable alleles originating from both parents. This has been confirmed by the fact that the QTL identified for GYS, TKW, and GPC came from both parents (**Tables 1**–**5**).

Although wheat is an important source for protein, most of the modern varieties are relatively low in grain protein (10–14%) especially in cultivars containing the *Rht1* dwarfing gene (McClung et al., 1986). Breeding efforts to increase GPC have been influenced by the strong impact of environmental conditions and by the negative effect on yield components, especially when the alleles come from wild emmers (Simmonds, 1995; Feil, 1997; Oury et al., 2003; Oury and Godin, 2007).

In this study the evaluation of grain yield components and GPC in three field trials lead us to the identification of 22 QTL distributed among all chromosomes excluding 1A, 3B, and 6A. This confirmed previous studies where QTL for GPC and grain yield were identified on almost all wheat chromosomes (Börner et al., 2002; Marza et al., 2005; McCartney et al., 2005; Quarrie et al., 2005; Huang et al., 2006; Gadaleta et al., 2007; Kuchel et al., 2007; Kumar et al., 2007; Maccaferri et al., 2008; McIntyre et al., 2010; Marcotuli et al., 2017; Mangini et al., 2018). Several QTL were found significant only in a one environment, and several authors reported that when a QTL was detected in more than one environment, a variation in its effects occurred (Huang et al., 2003; Huang et al., 2004; Kumar et al., 2007; Kuchel et al., 2007; Maccaferri et al., 2008; McIntyre et al., 2010).

By comparing the GPC-QTL found in the present study with those reported in the most recent literature, we found that loci on 2BS, 4BL, and 5BL were also reported by Nigro et al. (2019) and they occupy almost the same genomic region. Moreover, QTL on 2B, 4A, and 7B were also found in the work by Marcotuli et al. (2017). On chromosomes 2B and 4A we found QTL on a different chromosome arm (2BS instead of 2BL and 4AL rather than 4AS). For what concerns 7B, different stable QTL for GPC have been reported in diﬀerent studies (reviewed by Kumar et al., 2018), most of which were located on the short arm of chromosome 7B. However, accordingly to what reported by Blanco et al. (2006) and Marcotuli et al. (2017), we found the QTL on 7B located on the long arm of the chromosome. Also in the work by Roselló et al. (2018) we found GPC loci on 4A, 4B, 5B, and 7B. Differences observed in map position could be due to the different mapping populations used for QTL analyses, the marker coverage of linkage maps, and the high number of genes controlling the trait.

Our work confirmed the negative relationship between GPC and yield component traits (**Table 2**), although only the QTL for GYS and GPC on 2B chromosome were co-localized. The other QTL on 3A, 4A, 4B, 5B, and 7B showing significant effects on GPC values were interesting because not liked to yield potential, probably because contain genes that influence GPC independently from variation in the grain yield components, and could be used to improve the GPC. The high-density consensus linkage map for durum wheat described by Maccaferri et al. (2019) was used as reference for chromosome localization and SNP markers position to compare our results with those reported in literature.

A total of 16 QTL for GYS and TKW were also detected on almost all chromosomes of durum wheat, of which none coincident. These data are consistent with several works that reported significant QTL for yield components almost on all wheat chromosomes (Araus et al., 2008; Cui et al., 2014; Gupta et al., 2017). QTL for TKW were found on 1B, 3A, 4B, 5A, and 5B chromosomes, while QTL for GYS were localized on homoeologous chromosome group 2, 4, 5, and 7 as also reported by Mangini et al. (2018) and Marcotuli et al. (2017), respectively for loci on 2BS and 4AL, and 2BS. Most of the yield loci mapped in the present work confirm the mapping data reported in recent literature: a very good correspondence was observed for the TKW-QTL detected on 1B (1A), 3A, 4B, 5A, and 5B which were also reported by Wang et al. (2012); Assanga et al. (2017) and Su et al. (2018). In particular, the TKW-QTL identified in the present work on chromosomes 1B, 4B, and 5B localized on the same map confidence interval of those mapped by Wang et al. (2012) and Su et al. (2018).

In the present work, beside surveying new genetic variability for GPC and yield components, some alleles related to grain storage protein composition were transferred from bread wheat to durum wheat. Glutenin subunits or their alleles can be used as indicators of wheat quality and can be used as "markers" for breeding purposes. In particular, we obtained durum lines with 1Ax2* allele encoding for 2* HMW-GS, and lines with alleles encoding 7 + 9 HMW-GS. Branlard and Dardevet (1985) observed that Zeleny sedimentation value had positive correlation with subunits 7 + 9 and 5 + 10, and negative correlation with 2 + 12; moreover, extensibility had relationship with subunits 2* and 17 + 18. γ-gliadins 45 and 42 are valuable markers for good and poor pasta quality, respectively, and this is because the genetic linkage with low molecular weight glutenin subunits (Sapirstein et al., 2007). In the present work, durum lines with γ-42 gliadin subunit have been obtained and the identification of such lines suggests the possibility to improve durum wheat for bread-making processing, which is a common practice in the bakery tradition of certain Italian regions.

In conclusion, in the present work an enlargement of genetic variability has been achieved in a RIL population obtained by the cross of a bread and a durum wheat line with different quality characteristics. Lines with higher protein content and/or composition could be usefully employed directly or as donor lines in future breeding programs. Moreover, for the two yield components, confident CGs were identified associated to the QTL on 5BL: *TTG* (Transparent testa glabra) for GYS, and *EF* (Elongation factor) for TKW. In both cases we can assume these genes as good candidates as they have been reported in literature to be correlated to seed storage proteins accumulation or determination of grain number per spike. In particular, TTG proteins have been well characterized in *Arabidopsis* and *Nicotiana*: they are involved in the regulation of several processes of plant development and immunity (Wang et al., 2009; Li et al, 2012). In this work, *TTG* was chosen as putative candidate gene for GYS because other than taking part to anthocyanin biosynthesis (Pang et al., 2009; Zhang and Schrader, 2017), plant thricome formation (Morohashi et al., 2007; Wang et al., 2009), and pathogen resistance (Wang et al., 2009; Truman et al., 2010; Li et al., 2012), it was recently reported to be involved in seed storage proteins accumulation in *Arabidopsis* (Chen et al., 2015) and *Nicotiana* (Zhu et al., 2013; Ge et al., 2016).

For what concerns *EF*, in the work by Zheng et al. (2014) *Elongation Factor* expression has been reported to increase gradually during common wheat grain ﬁlling, expecially in young spikes and developing seedlings. Moreover, *Arabidopsis* transgenic plants harbouring a *TaTEF-7A* gene construct, showed a better vegetative growth and an increased silique length, silique number, and grain length. *TaTEF-7A* was also mapped in wheat using a DH population, resulting in a co-localization with several reported QTL for yield-related traits (spikelet number per spike, flour yield, test weight, and grain yield).

In conclusion, both candidate genes identified in this work well represent a good starting point for dissecting the molecular basis of some yield-related traits, and understanding the genetic mechanism underlying this complex quantitative trait. Moreover, they may also serve as useful tools for developing genetic markers suitable for MAS breeding programs.

## Data Availability Statement

All datasets for this study are included in the article/**Supplementary Material**.

## Author Contributions

AgG designed the experiments. SG performed phenotypic characterization of wheat lines for protein content and yield components. SZ performed protein extraction and electrophoretic evaluation. AnG performed QTL analyses and candidate gene identification. AgG and AnG collaborated to data interpretation and writing the manuscript. All authors read and approved the ﬁnal manuscript.

## Conflict of Interest

The authors declare that the research was conducted in the absence of any commercial or financial relationships that could be construed as a potential conflict of interest.
